# Detection-Response Task—Uses and Limitations

**DOI:** 10.3390/s18020594

**Published:** 2018-02-14

**Authors:** Kristina Stojmenova, Jaka Sodnik

**Affiliations:** Faculty of Electrical engineering, University of Ljubljana, Tržaška cesta 25, 1000 Ljubljana, Slovenia; kristina.stojmenova@fe.uni-lj.si

**Keywords:** detection-response task, cognitive distraction, driving, cognitive load, sensors, attentional resources

## Abstract

The Detection-Response Task is a method for assessing the attentional effects of cognitive load in a driving environment. Drivers are presented with a sensory stimulus every 3–5 s, and are asked to respond to it by pressing a button attached to their finger. Response times and hit rates are interpreted as indicators of the attentional effect of cognitive load. The stimuli can be visual, tactile and auditory, and are chosen based on the type of in-vehicle system or device that is being evaluated. Its biggest disadvantage is that the method itself also affects the driver’s performance and secondary task completion times. Nevertheless, this is an easy to use and implement method, which allows relevant assessment and evaluation of in-vehicle systems. By following the recommendations and taking into account its limitations, researchers can obtain reliable and valuable results on the attentional effects of cognitive load on drivers.

## 1. Introduction

Distracted driving is one of the major causes of road accidents. The National Highway Traffic Safety Administration (NHTSA) reported that almost 400,000 people were injured in motor vehicle crashes due to distracted driving in 2015 in the United States [1]. Due to its complexity as a psychological construct, driver distraction does not have one uniform definition and explanation. One of many definitions [2,3] of driver distraction states that “driver distraction is the diversion of attention away from activities critical for safe driving toward a competing activity” [4]. With regard to attention, which James defined as “concentration on a specific source of information” [5]; Recarte and Nunes suggest that driver distraction can be exogenous or endogenous [6]. Exogenous distraction is produced by external objects or events irrelevant to driving, whereas endogenous is produced by the driver’s cognitive activity unrelated to the task of driving (lost in thought or solving problems unrelated to the ongoing task of driving). Visual (eyes off the road) and manual (hands off the steering wheel) distractions are examples of exogenous distraction. Visual distraction has been tackled by changing the placement of information presentation, for example, head-up displays instead of classic head-down displays. Manual distraction can occur as a result of operating a mobile device or an in-vehicle information system (IVIS). A review of the trends in fatalities from distracted driving, reported by authorities in the United States, showed that there was a rise of 28% in fatalities due to texting and use of mobile devices in 2008 compared to 2005 in the United States [7]. These and similar reports explain the call for prohibition of cell phone use in many countries around the world. While visual and tactile distraction is due to sensory information perception and communication, driver cognitive distraction occurs as a result of the processing of information unrelated to driving. Cognitive distraction occurs as a result of increased cognitive load unrelated to the task of driving. Cognitive load can be defined as a multidimensional construct representing the load that performing a particular task imposes on the learner’s cognitive system [8]. In the ISO standard on the Detection-Response Task relating to driving, cognitive load is described as demand for higher level cognitive operations such as planning, decision making, error detection, sustaining information in the short-term (working) memory, and overcoming habitual actions [9]. All these operations can occur due to the processing of information when performing visual-manual tasks, conversing using a hands-free device, or by simply thinking of tasks irrelevant to operating the vehicle, and as such represents a great challenge to the research community. Although one may not be able to persuade drivers to stop thinking about work while driving, interface designers can increase IVIS or mobile device usability by presenting information in a way that it is less cognitively demanding. Nevertheless, this should be done with care so that an increase in IVIS usability does not have a counter effect: drivers get further intrigued and encouraged to use these systems due to a better user experience resulting in an even higher cognitive distraction.

This reveals the need for assessing the driver’s cognitive load and its impact on the driver’s mental state. It is also important to evaluate the amount of cognitive load imposed by different types of IVISs, which could result in cognitive distraction. This information can then be used already in the early stages of interface design in order to test and evaluate IVIS for cognitive distraction, with the purpose of achieving less cognitively demanding information exchange between the driver and the IVIS. Furthermore, attention and motor resources different than the ones needed for successfully operating the vehicle could be used. In addition, smart systems that can gain situational awareness of the driving environment and the driver’s mental state could also be introduced. They could warn the driver in case of cognitive (or any other) distraction or turn on drive assist systems, such as lane and park assist or autonomous cruise control. 

This paper offers a review of the Detection-Response Task (DRT)—a method for the assessment of the effects of cognitive load on driver’s attention based on a secondary task performance, where the secondary task is defined as a task that is not related to the performance of the primary task of driving [9]. We present its origins, areas of use and limitations, and also list a number of alternatives that can be used for the assessment of the driver’s cognitive load. 

## 2. Detection-Response Task

The Detection-Response Task is a method that evaluates the performance of a secondary task to observe the attentional effects of cognitive load. The method suggests that increased cognitive load would reduce the driver’s attention to other visual, tactile or auditory information, and thus result into the driver missing and not answering the presented DRT stimuli. Drivers are presented with a sensory stimulus every 3–5 s, and are asked to respond to it by pressing a button attached to their finger. Response times and hit rates are interpreted as indicators of the attentional effect of cognitive load. Response times are measured as the time from stimuli onset until the time the driver responds to it, and hit rates are calculated as the ratio of correctly answered stimuli (from 100 ms to 2500 ms) out of all presented stimuli. It is therefore important to choose the right stimuli modality and stimuli placement so that they are always detected, and not masked by the environment or missed due to driving related tasks. In order to assess the imposed load of a specific secondary task, a DRT measurement is performed independently without this task and simultaneously with the task. Larger differences in response times between the two conditions and lower hit rate ratios indicate higher cognitive distraction (load imposed by the task).

### 2.1. Factors Influencing the Response Time

**Figure 1:**
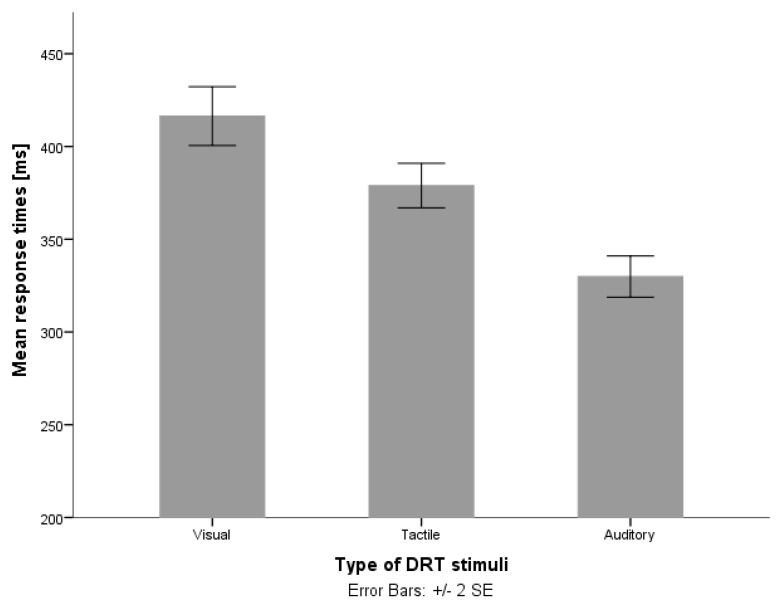
Mean response times for visual, tactile and auditory stimuli [10].

The stimuli can be visual, tactile or auditory, depending on the secondary tasks (systems) that are evaluated, and the environment in which the study is conducted. In order to achieve higher sensitivity of the method (to detect also smaller changes in cognitive load), it is convenient to find a signal that would evoke fast response times without an additional task and, on the other hand, evoke significantly longer response times when exposed to increased cognitive load. In Annex E, the summary of results from the ISO coordinated studies [9], and a study performed by Stojmenova and Sodnik [10] for trials performed without any additional secondary tasks, it was shown that auditory stimuli evoke the fastest response times compared to tactile and visual stimuli, and tactile stimuli evoke a faster response than visual stimuli (Figure 1). 

This is somewhat expected as (all being equal) response to light has a longer latency compared to sound or touch [11]. This is due to the fact that sound signals reach the brain in 8–10 ms [12], while light signals require from 20 to 40 ms [13]. Respectively, response times for touch are somewhat in-between at 155 ms [14], for tasks where answering stimuli is the primary and only task.

Furthermore, Chocholle explored how stimuli intensity affected response times. He revealed that for pure tones the response time decreases with increased signal intensity [15], but only until approx. 100 dB. The response time at 100 dB is approx. 109 ms [16], which is defined as the irreducible minimum by Chocholle, and above this limit, the sound intensity does not have any effect on response times. Additionally, Niessen showed that increased intensity also affects alertness and decreases the time needed to direct attention to a stimulus [17]. 

This shows that special precaution should be taken when comparing different studies using the DRT. The method’s sensitivity can be manipulated by increasing the stimuli intensity in order to reduce the basic response time (trials without a task), and hence increase the difference in response times compared to trials with a task [18], for example in environments with a bad signal-to-noise ratio. Preferably the same stimuli modality and intensity should be used when comparing different IVISs or relationships between different types of in-vehicle interaction and cognitive load in order to achieve reliable cross-cite studies and analysis.

### 2.2. DRT Versions and Types of Stimuli

The versions using visual and tactile stimuli were standardised by ISO in 2016 [9]. The standard gives guidelines for two possible placements of visual stimuli, head-mounted (HDRT) and a remote (RDRT). The latter version can sometimes be referred to as the Peripheral Detection Task (PDT), which is actually the method DRT was developed from [19,20,21]. In the standardised DRT version, visual stimuli can be placed in the driver’s peripheral field of view, usually on the left bottom side of the windshield or on the top right side of the dashboard. The standard proposes the use of a single red light source presented with an LED. In the case of a driving simulator environment, a graphical object is displayed at a fixed location on the visual display [9].

**Figure 2:**
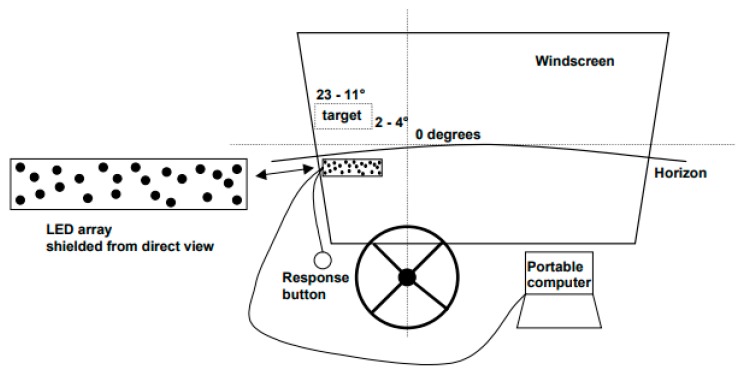
Peripheral Detection Task set-up [21].

The PDT method, on the other hand, is used primarily for assessing the driver’s visual distraction during driving [21]. It therefore provides more alternatives for the placement of the stimuli. It can be placed in the peripheral field of view, in the form of a fixed set-up or interchangeably—the location of the source of light would change for each next stimulus [22]. For example, one (randomly chosen) out of 23 available light sources would light up (Figure 2) [21], a stimulus would change position throughout the experiment from left to right or in a randomly chosen direction [20]. 

It is important to note that the PDT does not measure the size (width) of the functional field of view (visual tunnelling) [22], and should be used exclusively for assessing the effects of visual distraction and, consequently, cognitive load. Its main disadvantage and limitation is the use of a visual stimulus placed in the driver’s peripheral field of view. Regardless of the position of the stimulus, drivers can overlook and miss it while turning their head, looking at the rear mirrors or due to any other visually demanding driving tasks, and not because of increased cognitive load. 

**Figure 3:**
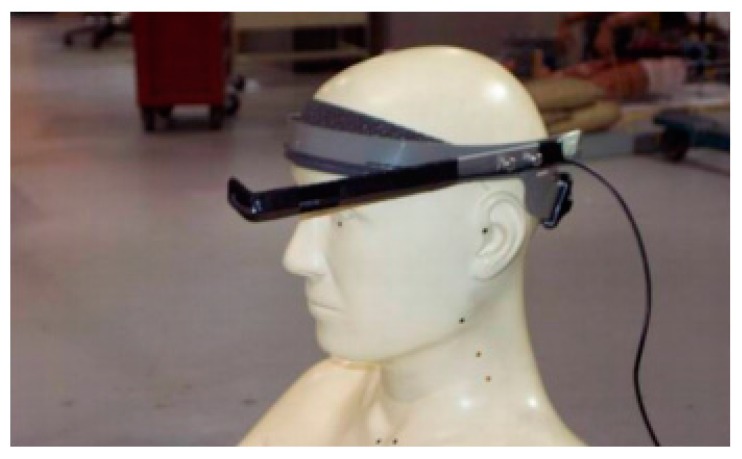
Head-mounted Detection-Response Task (HDRT) set-up [23].

The other standardised visual version, HDRT, solves this problem by head-mounting the stimuli source so that it always stays in the field of view, regardless of the driver’s head position. With this version, the visual stimuli are presented in front of the driver’s left eye staying visible at all times (Figure 3). It is important to note that the DRT method is used to evaluate the effect of cognitive load on the driver’s attention and resource allocation imposed by the performance of secondary tasks. The latter mainly represent the use of IVISs or other types of driver-vehicle interaction. At the moment, the majority of IVISs in vehicles display (output) information visually and are operated (input) manually (visual-manual systems). Human attention is limited, and a visual perception channel can only perceive a limited amount of information at a time [5]. The perception of visual DRT stimuli, reading information on the IVIS display and paying attention to the driving environment, which requires a significant amount of visual attention, can therefore be overwhelming. Consequently, the use of the visual DRT in such circumstances can be inappropriate and can cause sensory overload. 

**Figure 4:**
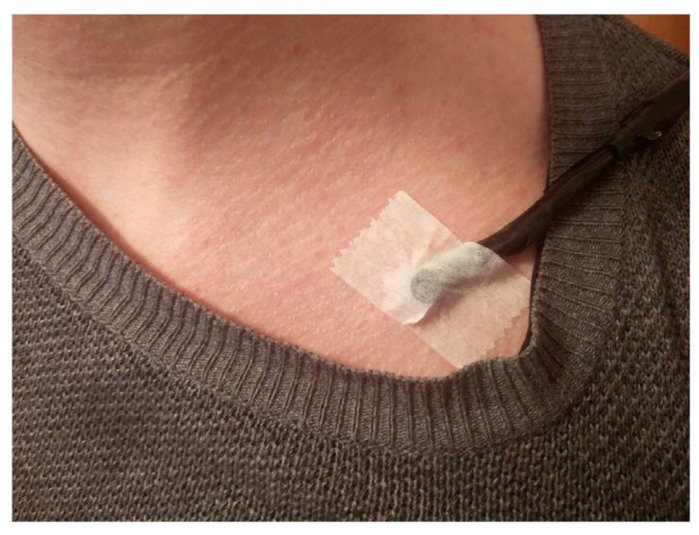
Tactile Detection-Response Task (TDRT) set-up [24].

In order to avoid sensory overload, tactile or auditory stimuli can be used instead. For example, the TDRT uses vibration as stimuli, produced with a small 10 mm vibrating tactor with a maximum speed of 12,000 rpm and vibration amplitude 0.8 G [9]. It is placed on the driver’s left shoulder collar bone for left-side operated vehicles, and on the right for vehicles with the steering wheel on the right side (Figure 4). Like with a visual DRT, the driver’s task is to respond to the stimuli as soon as perceived by manually pressing a button. Although this version avoids the overlap in the perception channel (i.e., visual stimulus and visual-manual IVIS interaction), an overlap in the output channel still occurs as the driver uses the same biomechanical channel of touch to perceive tactile stimuli and to answer them by manually pressing a button.

Due to this reason, Stojmenova et al. proposed an auditory version of the DRT (ADRT) using an auditory signal as a stimulus. In this case, there is no overlap with the visual or biomechanical channel [25]. Beforehand, the ADRT was also mentioned in studies by Merat et al. [26], Merat and Jameson [27] and Stojmenova et al. [25]. However, they all use different sound signals (beeps, broadband noise and modulated harmonic signals), which makes the direct comparison of the proposed methods almost impossible. The DRT standard also does not provide any guidelines for the selection or implementation of the most appropriate auditory stimuli. Stojmenova et al. proposed a candidate for the auditory stimulus through a user study considering some general properties and limitations of the human hearing system. They took into consideration that the hearing system is only sensitive to stimuli containing from around 20 Hz to around 20 kHz, and is the most sensitive to frequencies between 2 kHz and 5 kHz [28]. Their results were most favourable towards 4 kHz and 8 kHz pure tones, played at 53 dB. Finally, they recommend the use of 4 kHz with a slightly increased intensity, primarily due to the fact that the upper limit of hearing decreases with age and therefore the 8 kHz could be more difficult to perceive for older drivers. They later validated their choice by performing a study in which they compared their ADRT version (4 kHz stimuli played at 60 dB), with the standardized RDRT and TDRT, and showed that the ADRT is also sensitive to the attentional effects of cognitive load [10]. 

### 2.3. Choosing the Right DRT Version (Relationship between the DRT Version and the Secondary Task)

As indicated before, all DRT versions are used to assess the attentional effects of cognitive load imposed on the driver by secondary tasks. There is a strong relationship between the type of secondary task and the DRT version used due to the previously presented potential overlap of input/output channels (i.e., human senses and responders). The two most commonly used groups of secondary tasks in vehicles are:

- visual-manual tasks: typical interaction with in-vehicle infotainment systems (IVIS) where the information is shown visually and the device is operated manually (e.g., head-down or head-up displays operated through a touchscreen or various buttons and levers in the cockpit);
- pure cognitive tasks: in-vehicle tasks where information is presented auditorily and responses are given vocally (e.g., navigation device operated through speech commands or simple phone conversations based on hands-free systems).

These two types of typical in-vehicle tasks can be simulated by two experimental tasks enabling a high level of controllability and experimental validity.

**Figure 5:**
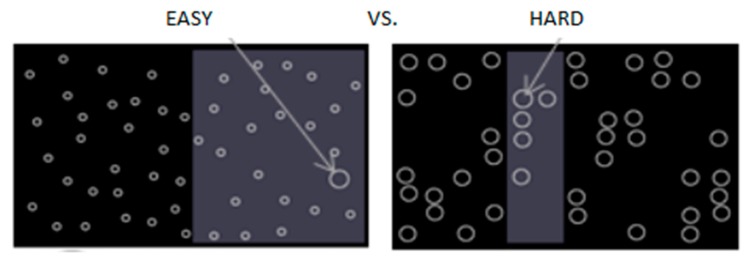
Visual-manual Surrogate Reference Task (SuRT) [30].

The Surrogate Reference Task (SuRT) is a standardized visual-manual task where drivers are presented with a number of circles of the same size and one larger “target” circle. They have to point out the target circle using the left and right keypad buttons [29]. Depending on the difference in the size of the target circle and other circles, the level of demand can be easy or hard (Figure 5). 

Delayed digit recall is a pure cognitive task, also known as a modified n-back task [31]. It is a simple task where drivers are presented with spoken auditory stimuli in the form of single-digit numbers, which they have to repeat back to the experimenter following specific rules. The structure of the task allows several difficulty levels: mild, moderate and high level of task demand. For the mild level, the driver has to repeat immediately the last number presented—0-back, for the moderate the number next-to-last (1-back) and for the high level the number second-to-last (2-back). An example of the consecutive order of numbers and drivers’ responses is presented in Table 1. 

The sensitivity, to both visual-manual and pure cognitive secondary tasks, for all four versions of DRT methods has been evaluated in various studies [10,22,23,25,26,27,30]. Based on the results of these studies, the ISO DRT standard suggests primarily visual and tactile DRT variants to be suitable and sensitive for assessing distracting factors of audio-vocal systems in vehicles [9]. The tactile version should preferably be used for the assessment of systems that present information visually, and therefore require more visual attention. 

**Figure 6:**
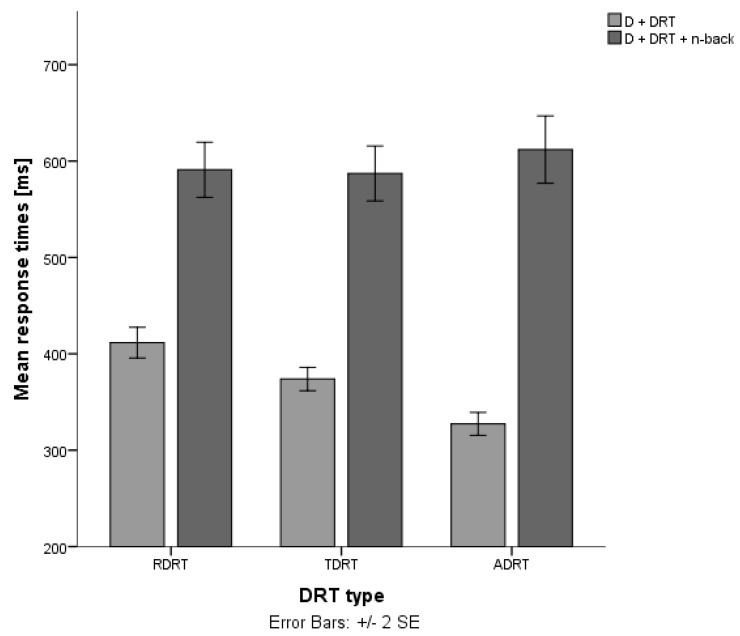
Response times for remote visual DRT (RDRT), tactile DRT (TDRT) and auditory DRT (ADRT) for trials without and with a cognitive task (auditory-vocal n-back task) [10].

Stojmenova and Sodnik, on the other hand, demonstrated that the audio version of the DRT also showed the greatest differences in response times when comparing trials without and trials with a cognitive auditory-vocal task (Figure 6) [10]. In their experiment, the same amount of cognitive load was induced across all trials with a cognitive task, therefore, the obtained differences in response times directly represent the sensitivity of each method. The results even indicate that the ADRT seems to be the most sensitive version of the DRT methods for assessing the impact of this type of secondary tasks.

### 2.4. Response to the Stimulus

**Figure 7:**
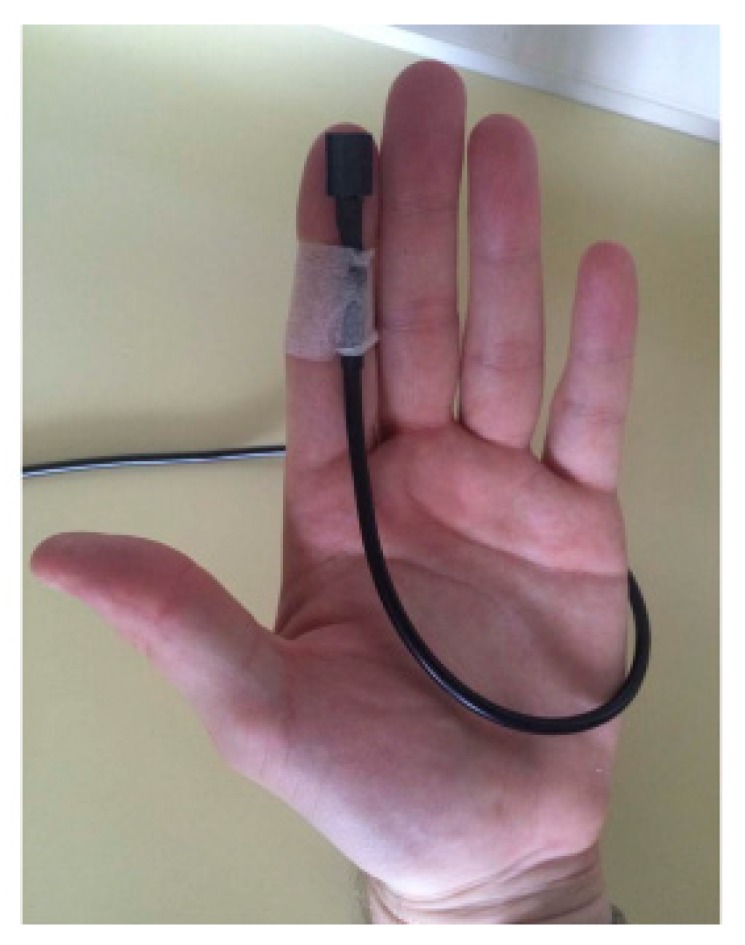
Response button used for answering to Detection-response task (DRT) stimuli [24].

The response method has also evolved through the years. In the first research that mentions the RDRT (PDT), a verbal response was used to indicate that the stimuli were perceived [32,33]. In surrogate driving environments, where drivers, instead of driving, watch a driving video recorded from a driver’s perspective, tapping on the pedal brake was also used as a response method [34,35]. Nevertheless, the most common and now standardised response method is a manual response based on a button press [9,10,20,30]. The ISO standard recommends that a button is attached to the thumb, index finger (Figure 7) or, if requested, any other finger, on the left hand for left-operated vehicles and vice versa, and it is pressed against the wheel to respond to the presented stimuli [9]. 

Paul Fitts discussed the importance of “compatibility” of the stimulus and response in terms of their modality (e.g., visual-vocal, visual-manual, auditory-manual, tactile-manual, etc.) [36]. The properties of the relationship between the two turned out to be as important as their individual properties. It refers to the finding that different combinations of stimuli and response modalities produce different results (e.g., faster and more accurate responses) [37]. This raises a question about the possibility of defining a standardised answering method for the DRT as currently all four versions of the method use the same response method.

### 2.5. DRT Intrusiveness

An important point to be considered when using this sensory based method is the fact that although it tries to capture the effects of secondary tasks on the driver’s attention, the method itself is also a secondary task, and as such distracts the driver from the primary task. Regardless of the stimuli modality, the DRT requires constant driver involvement and diverts his/her attention from driving to detecting the presented stimulus. Stojmenova and Sodnik conducted a study in which they explored the intrusiveness of the method by observing changes in three dependent variables [38]:

- psychophysical response (i.e., pupil dilation),
- driving performance (i.e., acceleration and lane deviation/departure), and
- secondary task performance (i.e., performance of the secondary cognitive task, success rate and task completion times).

In the study, participants performed eight trials:

- driving
- driving and a cognitive task (n-back task)
- driving and RDRT
- driving and RDRT, and a cognitive task (n-back task)
- driving and TDRT
- driving and TDRT, and a cognitive task (n-back task)
- driving and ADRT
- driving and ADRT, and a cognitive task (n-back task).

**Figure 8:**
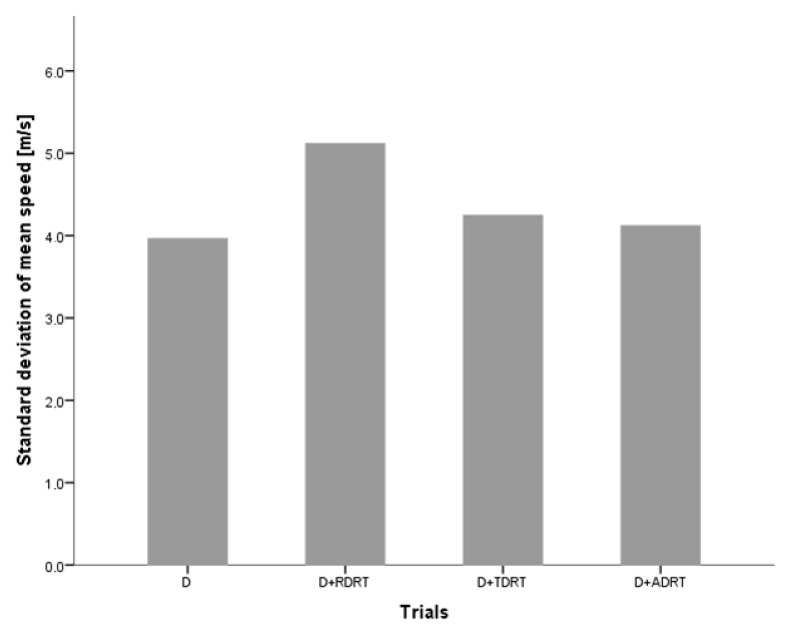
Standard deviation of the mean speed across trials without and with visual, tactile and auditory DRT stimuli [38].

**Figure 9:**
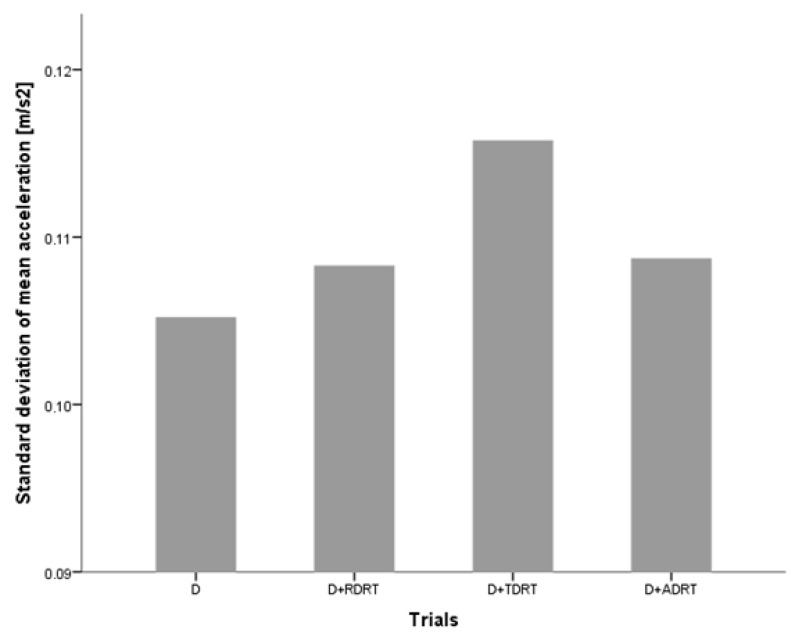
Standard deviation of the mean acceleration deviation across trials without and with visual, tactile and auditory DRT stimuli [38].

**Figure 10:**
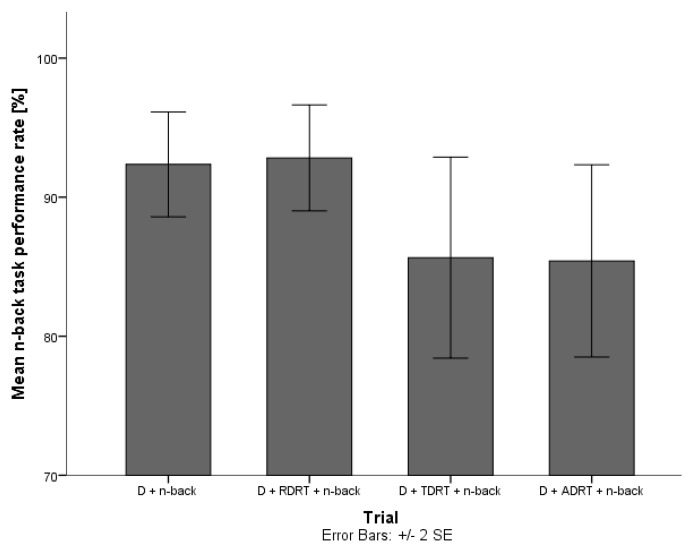
Mean task performance rate for trials without and with DRT stimuli [38].

**Figure 11:**
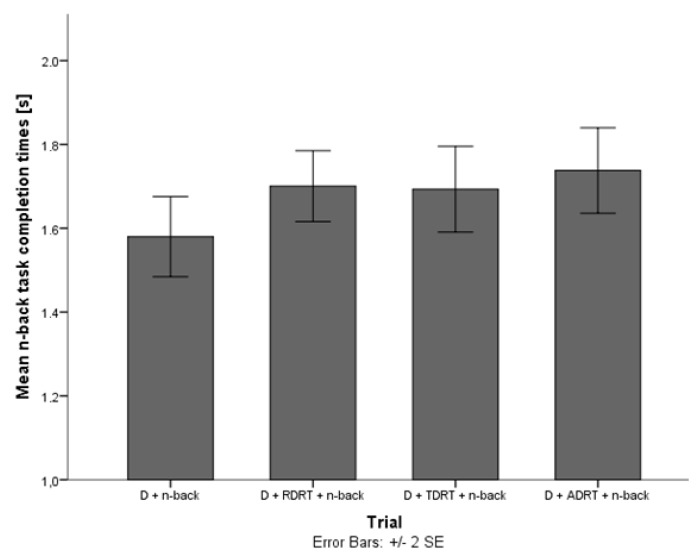
Mean task completion times for trials without and with DRT stimuli [38].

They explored how these three variables changed in the presence of DRT stimuli. The driver’s pupil size data for trials without the DRT and trials with the DRT did not show significant differences, indicating that answering DRT stimuli does not impose additional cognitive load on the driver. Results on acceleration deviation and task completion times suggest that answering DRT stimuli affects driving (Figure 8 and Figure 9) and secondary task performance (Figure 10 and Figure 11).

#### 2.5.1. DRT Intrusiveness on Driving Performance

In the study, participants were asked to drive at a constant speed of 130 km on a simulated highway route with low traffic intensity, which did not require them to overtake any vehicles. Consequently, the average driving speed did not change much during different trials, and standard deviationof the speed increased significantly when comparing trials without answering the DRT and trials with the RDRT and TDRT (Figure 8). There were also significant increases in the standard deviation of mean acceleration for trials with all three versions of RDRT, TDRT and ADRT compared to trials without DRT stimuli (Figure 9).

When performing more than one task, drivers can adjust their performance and allocate more effort to the primary task of driving [3]. In the study [38], this is evident from the fact that the average speed across all trials did not change; however, there was an increase in the average speed and acceleration deviations for trials with the DRT, indicating that participants had to put more effort into completing the task of driving at a constant speed. These results could be taken into consideration when evaluating IVIS with the DRT and driving performance data; some of the speed and acceleration deviations (if these are considered as driving performance indicators) could be the result of answering DRT stimuli and not necessarily because of the use of the tested IVIS. Additional caution should also be taken when performing studies with the DRT in a real driving environment, to ensure the test driver’s safety.

#### 2.5.2. DRT Intrusiveness on Secondary Task Performance

The performance of the secondary task (i.e., a delayed digit recall task (n-back task) [31]), did not decrease statistically significantly when users were asked to respond to the DRT compared to trials without the DRT. However, an obvious fall in the performance success rate is evident for trials with the TDRT and ADRT (Figure 10). Furthermore, the time needed to complete each part of the task increased significantly for trials which also included answering DRT stimuli (Figure 11). As the task completion time is a common indicator of a system’s usability, researchers should be careful in interpreting this data when evaluating a system using the DRT.

## 3. Alternative Methods for the Assessment of Driver’ Cognitive Distraction

Cognitive load does not have a quantity unit, and is therefore always observed relatively, mainly through other parameters that change when cognitive demand increases. It has been conceptualized as the allocation of mental resources or effort needed to perform one or more tasks at a time [39], and as a multidimensional construct representing the load that performing a particular task imposes on the learner’s cognitive system [8]. As shown with the DRT, cognitive load and its effect on attention can be successfully measured by observing secondary tasks performance indicators, such as response times (time completion times) and hit rates (performance success). Driving performance is also observed as an indicator of changes in cognitive load [40,41,42]. One such method that uses degradation of driving performance is the Lane Change Task. With this method, drivers have to change lanes according to specific visually presented signs within a simulated driving environment. The number of successful lane changes, lane deviations, and steering angles when performing the lane change are compared for trials without and with a secondary task, to explore changes in cognitive load. However, Engström et al. suggest that cognitive distraction in this method only affects detection and response to the visual signs (successful lane changes), whereas lane deviations and other driving performance indicators are more a result of visual distraction [43]. Unlike the DRT, this method can only be used in simulated driving environments, which are usually very simple and do not include any other traffic interaction in order to not compromise the primary task of lane changes. This raises the question of the ecological validity of studies performed with this method or whether the obtained results from such experiments can be replicated in real-life situations.

Alternatively, an increase in cognitive load can also be reported subjectively through self-assessment questionnaires or by monitoring psychophysiological parameters, such as pupilometry or cardiovascular, electrodermal and neural activity. 

Many studies use self-evaluation questionnaires to determine the level of cognitive load that a particular task may impose on an operator. Although self-ratings may appear questionable, studies have demonstrated that people are capable of giving a numerical indication of their perceived load [8]. However, some information can be lost or affected due to the nature of collecting data, which is always after completing the task. At that point the participant could have already forgotten (if the task lasts a longer period of time), or could be influenced only by the events towards the end of the task (the last thing the participant remembers). Nevertheless, self-evaluation questionnaires are commonly used due to their cost and time efficiency and relatively simple administration. 

Most subjective measures are multidimensional in that they assess groups of associated variables, such as mental effort, fatigue, and frustration, which are all highly correlated [8]. For example, the NASA Task Load questionnaire (NASA-TLX), in the original [44] and numerous modified forms [45,46,47], is one of the most common and still widely used scales for self-assessment. As a multi-dimensional rating procedure, it provides an overall workload score based on a weighted average of ratings of originally six dimensions: mental, physical and temporal demand, and performance, effort and frustration. The Subjective Workload Assessment Technique (SWAT) uses only three: time, mental and psychological stress load, and similarly to the NASA-TLX it uses conjoint measurement and scaling techniques to develop a single, global rating scale [48]. This rating score, however, does not give information only on cognitive load; it is an overall assessment of the driver’s workload for performing a task, which can also be due to the visual, manual or other type of demand. Both methods have been primarily used for the assessment of aircraft operators but have since spread also to other vehicle operators. 

Furthermore, for more detailed and extensive research, questionnaires such as the Workload Profile (WP) are used. The WP does not ask participants to rate the amount of load they have experienced, but to identify which attentional resource (the sheet of definitions on each of them is also provided) they had to use to complete the task [49]. This method has been shown to be superior in sensitivity compared to the NASA-TLX and SWAT [50], and due to the fact that it does differentiate between resources, cognitive load can be distinguished from other types of resource demand. Nevertheless, its implementation and interpretation are much more challenging than the former two. Although questionnaires represent a relatively easy way of gathering data, they are often considered as unreliable and are usually accompanied with at least one other method for workload assessment. This suggests that they are used more as a complementary method to the DRT, rather than as an alternative.

Observing and capturing psychophysiological parameters, on the other hand, is quite the opposite. It requires higher financial and technical input, but it is usually viewed as more valid and reliable. There are a number of methods and sensors developed for assessing the physical and psychological state of the driver in relation to his/her cognitive load. Heart rate (HR) sensors, for example, are one of the earliest and commonly used biometric indicators of changes in cognitive load in vehicle operators [51]. Heart rate variability (HRV) is used, with a number of researchers indicating that it is superior to HR [52] when assessing changes in cognitive load. Mehler et al., however, suggest that both parameters can be used as indicators of changes in cognitive load. In their study, results even indicated that with HR it is possible to detect also a low-level task, unlike HRV, which was only sensitive to higher demands of cognitive load [53]. Still, the authors comment that they do not support the idea that one measurement is superior to the other. Instead, they suggest that physiological measurements can be influenced by the conditions and environment in which the testing is taking place, as well as the type of workload that is imposed by the secondary task (for example, pure cognitive or not). They also suggest supporting cardiovascular measurements with electrodermal measurements, such as skin conductance, to further increase the reliability of such tests. Electrodermal activity (EDA) sensors are used to collect electrophysiological data to explore and understand the emotional and cognitive process. It is important to note that EDA is composed of two components: the electrodermal level, which occurs as a thermoregulatory process, and electrodermal responses, which are correlated with sympathetic nervous discharges [54]. The latter are interpreted as indicators of a particular emotional or cognitive faction. As mentioned earlier, skin conductance is used as a supporting measurement for the assessment of the driver’s cognitive load [53,55]. We could not find any studies that would report on the driver’s cognitive load or cognitive distraction relying solely on this measurement. Furthermore, it is important to be aware that the same sweat glands are also activated when humans experience stress, and models that differentiate between these two should be applied when interpreting the data [56]. 

As a substitute, eye activities such as pupil size, blink frequency and blink frequency variability can also be used for the assessment of a driver’s cognitive demand. Pupil size, for example, increases when humans are exposed to increased cognitive demand [57,58]. Researchers have reported studies in which the effects of cognitively demanding tasks while driving can be detected with both low-cost and high-end eye trackers [24,59,60]. Instead of only observing the physical changes in the driver’s eyes, it has been found that cognitive distraction can also be understood from visual behaviour, such as following the driver’s gaze (eye tracking) and effects (visual tunnelling and eye fixations), as it affects the driver’s visual field horizontally and vertically, and glances at the rear mirrors or speed signs are less frequent [6,61]. The biggest disadvantage of eye trackers is that the eye is very light sensitive, and the collected data can also be influenced by visual load and distraction. Therefore, eye activities, especially pupil size, can also be the result of changes in surrounding lighting or due to visual information related to traffic (signs, brake lights or traffic lights). In controlled driving environments, such as driving simulators, these difficulties can be avoided; however, in real-life vehicles, eye-trackers can be more difficult to use. 

Furthermore, electroencephalogram (EEG) is also used for the assessment of the driver’s cognitive distraction and can be interpreted as the closest sensory-based method to directly measure cognitive load. EEG variables that indicate changes in cognitive demand are alpha suppression, increased beta, increased frontal midline theta, and ratios such as beta–alpha plus theta and alpha plus theta–beta [62,63]. While the EEG can record even the slightest increase in cognitive load, it cannot detect cognitive underload, which can occur due to the lack of attention [64]. For this type of cognitive distraction, event-related potentials (ERPs) are more appropriate. The ERP is a direct brain response to sensory, cognitive or motor stimuli, and is also recorded with EEG sensors. Most of these methods were developed for the assessment of cognitive load in general, and not solely for the assessment of drivers’ cognitive load and distraction. The driving environment is quite dynamic and the testing conditions are much different than laboratory and research noise-free and controlled ones, which calls for precaution when interpreting the data, or use of a much simpler solution (if the study characteristics allow it), such as the Detection-Response Task. 

## 4. Conclusions

The detection-response method (DRT) has gained popularity in the human-computer interaction (HCI) research community mainly due to its simple implementation, lower cost than use of biometric sensors, and more reliable detection of the effects of cognitive load compared to self-evaluation questionnaires. The need for such a method has also been confirmed by all the suggestions for improvements during the years and the effort invested into developing four different versions with the purpose of making it suitable for different testing environments and various IVIS modalities. 

In order to collect reliable data, the method characteristics and limitations should be taken into consideration when performing research studies, and the right version should be chosen based on the environment and the tested in-vehicle interaction and infrastructure. The DRT relies on the perception of either visual, tactile or auditory stimuli, which can cause an overlap in modalities used for interaction with in-vehicle interfaces. These overlaps should therefore be avoided when selecting the most appropriate DRT method. For example, the visual versions, RDRT or HDRT, could be used for testing less visually demanding IVISs as they all rely on the same perception channel. Similarly, the tactile version, TDRT, should not be used for interfaces relying on tactile information presentation (for example seat or steering wheel vibrations), and could be used instead for visual or auditory interfaces. The auditory DRT could be used for the assessment of pure cognitive tasks and visual-manual interfaces. From the reported evaluation of various research studies, it can be concluded that different modalities also enable the use of different testing environments; for desktop simulators, where head turning is not required, the RDRT offers the simplest implementation. The ADRT or TDRT, on the other hand, could be used for set-ups with changing lighting or a number of visual distractions, such as traffic and vehicle lights, dash-board information signals or direct sunlight, which could lead to overlooking visual stimuli. 

It is important to mention that although the DRT method does not impose additional cognitive load, it does affect the driver’s performance and secondary task performance. Increased speed and acceleration variations indicate that the driver’s performance is affected by the DRT, which on real roads can endanger the driver and the rest of the traffic participants. Controlled driving environments, such as simulators and surrogate driving set-ups, may be more appropriate when using this method in order to avoid casualties and preserve test drivers’ safety. Task completion time is a common indicator of a system’s usability and intuitiveness. Because answering DRT stimuli increases the task completion times for secondary tasks (operation of IVIS), researchers should be careful when interpreting these data.

Even though the implementation methods and collection of response times can be performed with an average PC, its implementation was made even easier after Krause et al. published an open source code and detailed step-by-step instructions on how to construct an Arduino based system for stimuli presentation and collection of response times [65]. Alternatively, there are also available ready-to-use DRT sets [66], or even companies that offer performing DRT studies as a service [67].

After reviewing the potential uses and limitations of the method, and comparing it with the possible alternatives, the detection-response task can be considered as an easy to use and implement method, which allows relevant assessment and evaluation of in-vehicle systems. By following the standard and other published recommendations for different environments, while taking into account the method’s limitations and intrusiveness, researchers can obtain reliable and valuable results on the effect of cognitive load on drivers’ attention.

## Figures and Tables

**Table 1:** N-back task in high level—2-back task.

	Consecutive Order of Digits									
Stimuli	3	9	7	4	6	5	5	1	8	9
Response	-	-	3	9	7	4	6	5	5	1
